# Association between real-time strategy video game learning outcomes and pre-training brain white matter structure: preliminary study

**DOI:** 10.1038/s41598-022-25099-0

**Published:** 2022-12-01

**Authors:** Paulina Lewandowska, Natalia Jakubowska, Nikodem Hryniewicz, Rafał Prusinowski, Bartosz Kossowski, Aneta Brzezicka, Natalia Kowalczyk-Grębska

**Affiliations:** 1 Institute of Psychology, Jagiellonian University, Romana Ingardena 6, 30-060 Krakow, Poland; 2 Faculty of Psychology, SWPS University of Social Sciences and Humanities, Chodakowska 19/31, 03-815 Warsaw, Poland; 3 CNS Lab, Nalecz Institute of Biocybernetics and Biomedical Engineering, PAS, Księcia Trojdena 4, 02-109 Warsaw, Poland; 4 Laboratory of Brain Imaging, Nencki Institute of Experimental Biology, PAS, Ludwika Pasteura 3, 02-093 Warsaw, Poland; 5 Polish-Japanese Academy of Information Technology, Koszykowa 86, 02-008 Warsaw, Poland

**Keywords:** Psychology, Neuroscience

## Abstract

In recent years the association between video games, cognition, and the brain has been actively investigated. However, it is still unclear how individual predispositions, such as brain structure characteristics, play a role in the process of acquiring new skills, such as video games. The aim of this preliminary study was to investigate whether acquisition of cognitive-motor skills from the real-time strategy video game (StarCraft II) is associated with pre-training measures of brain white matter integrity. Results show that higher white matter integrity in regions (anterior limb of internal capsule, cingulum/hippocampus) and tracts (inferior longitudinal fasciculus) related with motoric functions, set shifting and visual decision making was associated with better Star Craft II performance. The presented findings inline with previous results and suggest that structural brain predispositions of individuals are related to the video game skill acquisition. Our study highlights the importance of neuroimaging studies that focus on white matter in predicting the outcomes of intervention studies and has implications for understanding the neural basis of the skill learning process.

## Introduction

The development of the video game (VG) industry in recent years has had a significant impact on society. Statistics show that in 2021 there were around 3.0 billion players across the world, which means that the number of players grew by 5% within a year^1^ (Newzoo, 2021). Together with the growth in the popularity of VGs, there has been an increase in the body of literature investigating the association between gaming, cognition, and the underlying structure of the brain. Research describes playing VGs as a complex task because it requires practice to learn specific skills (e.g., visual, motor), engage various cognitive functions, and force rapid decision making^2,3^. Many cross-sectional studies prove that video game players (VGPs) outperform non-video game players (NVGPs, little or no experience with video games) in attentional and perceptual abilities, such as processing speed^4^ object orientation^5–7^, distribution of visual information^8^ and switching between stimuli^5,6^. In addition to that, there are few studies using neuroimaging methods reporting differences in the brain structure between VGPs and non-players. Players, compared to amateurs, exhibit a larger gray matter (GM) volume in the lenticular nucleus^9^, greater white matter (WM) integrity of the visual and motor pathways^10^, as well as increased structural connectivity between the frontal and occipital regions^11^, frontal and parietal regions and within the occipital regions^12^. Longitudinal studies using VGs as a training task in the group of non-gamers show the positive influence of VGs on the human brain^13–15^ and cognition^6,16^. However, available work, including meta-analyses discuss no causal relationship between VG playing and enhanced cognitive ability^17,18^ and suggest that the true effect of VG training on general cognition is close to zero^19^. In addition to the methodological issues with the studies included in these meta-analyses, it may be possible that VG training is more effective for some people and may be related to predispositions to a complex task learning. Theories based on individual differences go back to the 1950s, explaining that the skill learning process that makes people more efficient and accurate in specific tasks depends on personal characteristics. Kanai and Rees^20^ claim that brain structure is shaped through life by different factors such as upbringing, genetics, or environment, and that is why interindividual differences in brain structure might be a valid measure to predict differences in complex skills learning between people. Furthermore, a recent article on MRI predictors of cognitive training outcomes concludes that specifically WM integrity may provide information on who can benefit from different types of learning task and who can show higher cognitive performance after cognitive training^21^. WM microstructure characteristics are obtained with the diffusion weighted imaging (DWI) method that allows one to measure characteristics such WM integrity, and/or mapping of nerve fiber pathways^22^. One of the most commonly used measures of WM characteristics is fractional anisotropy (FA), which indicates the degree of anisotropy and is sensitive to neural properties such as axon myelination, and fiber density and diameter^23^.

There is still little body of literature that has discussed the neural predictors (white and gray matter characteristics) of VG performance. Available studies show that the volume of GM in the lenticular nucleus, composed of the putamen and the globus pallidum within the basal ganglia, significantly predicted the performance results of the real-time strategy (RTS) video game (StarCraft II) performance outcomes^9^. The study conducted by Erickson et al.^24^ investigated that striatal grey matter volumes predicted learning improvement of action VG (Space Fortress), where hippocampal volumes did not^24^. To our knowledge, there is one study focusing on the association between WM microstructure and VG performance outcomes^15^. The study by Ray et al.^15^ examined the above mentioned association in the context of two different genres of games, where easily accessible online games were chosen: “Sushi-Go-Round” (action video game), and “Tank Attack 3D” (strategy video game). They proved that the FA value, which refers to the integrity of the WM, predicted the performance outcomes for the action and strategy VG in different regions of the WM. FA in the left cingulum/hippocampus was correlated with strategy VG learning, while FA in the right fornix/stria terminalis was related to action VG learning.

In the presented study, we used an RTS game (StarCraft II) that is considered a great tool to study complex skill acquisition. In StarCraft II, players are asked to simultaneously gather, expand, protect the resources, micromanage single units within armies, and actions, where the orders might be executed immediately or put away for later. The literature suggests that StarCraft II is a cognitively demanding game that involves specialized skills such as rapid visual information processing, performing missions with precision in time, left and right-hand coordination, and transforming mental plans into motor movements^25^.

The recorded telemetry data from StarCraft II allows for extracting game indicators such as Perception Action Cycles, Actions Per Minute, or hotkeys usage, that may reflect the improvement of cognitive and motor skills in the game (see Methods section).

In our study, we have recruited NVGPs who learned to play a complex RTS game—StarCraft II. Our aim was to verify whether measures from white matter regions and tracts (obtained before the start of the video game training) are associated with the StarCraft II training outcomes. The white matter regions and tracts were chosen on the basis of the existing literature which applied different methods for analyzing the WM microstructure. We applied two approaches to analyze the data: in the first approach, we have extracted FA values for WM regions of interest, choosing them based on the previous research, whereas for the second approach, we have performed probabilistic tractography and calculated FA along the motor and visual pathways. The FA values within these pathways were shown to differ between VGPs and amateurs^10^, and our goal was to verify whether these tracts could be associated with VG skill acquisition.

## Methods

The data used in this study are part of the project ‘*The temporal dynamics of neurocognitive changes induced by complex task training in the form of strategic computer game*’ conducted at the NeuroCognitive Research Center (SWPS University in Warsaw) founded by the National Science Center. The research was approved by the Ethics Committee of SWPS University (number: 38/2018). All subjects signed an informed consent in accordance with Declaration of Helsinki before the first measurement session to participate in the study.

### Participants

**Figure 1:**
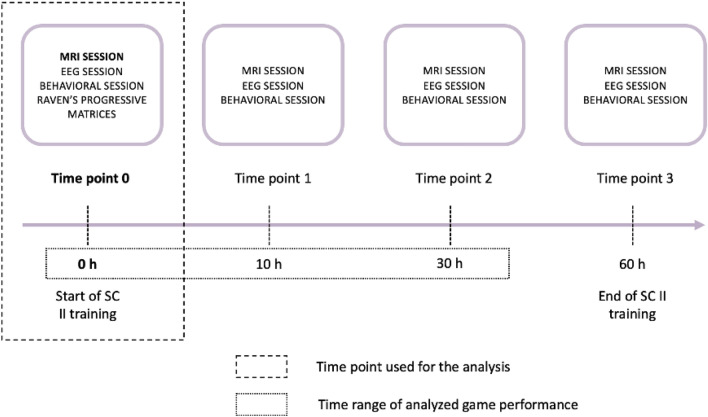
Graphic presentation of the study design. Data from a marked time range were used in this study. *MRI* magnetic resonance imaging, *EEG* electroencephalography.

In the whole study participants took part in the structural magnetic resonance imaging (sMRI) acquisition, cognitive evaluation, and EEG (electroencephalography) session at four time points: before VG training (T0), after 10 h of training (T1), after 30 h of training (T2) and after 60 h of training (T3). The presented study is focused on pre-training (TP0), diffusion magnetic resonance imaging (dMRI sessions) and the first 30 h of VG training (Fig. 1).

A total of 23 subjects that performed approximately 30 h (± 3 h) of StarCraft II training and completed the 3rd time point measurements (T3) were included in this study. One participant was excluded due to issues with dMRI images, and the final sample consisted of 22 participants (female = 15) with a mean age = 27.45 (SD = 5.12).

### Study procedure

First, subjects completed an online survey^26^ about VG playing experience, education, and demographics through the GEX platform (GEX Immergo, Funds Auxilium Sp. z o.o). The survey included questions about age, gender, level of education, marital status, work experience, total experience with video games in years, frequency of playing video games, devices used to play video games, game genres played by participants, subjective level of gaming expertise and the frequency of playing specific game genres. Participants' inclusion criteria were: [1] little or no previous experience with RTS video games, [2] experience with other types of video games with no more than 8 h/week during the last six months. All participants declared that they had no history of neurological illness, no use of psychoactive substances, and reported normal vision and hearing, and completed health-related questions prior to MRI sessions. Participants also carried out nonverbal group test, Raven's Progressive Matrices^27^ in order to control higher-order intellectual functions (mean score for all participants = 24.14, SD = 5.30).

Subjects recruited for the study were randomly assigned to two training groups: the experimental ‘*Variable’* group (n = 13, Mage = 27.1, SD = 3.52, 10 females) and an active control group ‘*Fixed’* (n = 9, Mage = 28, SD = 7.05, 5 females).

The groups differed on the basis of the opponent's race and strategy for each match. Since the main focus in our study is on the overall game skill acquisition, we have combined these two groups into one. Furthermore, the sample size was not sufficient to perform a separate analysis.

### Training task—StarCraft II

**Figure 2:**
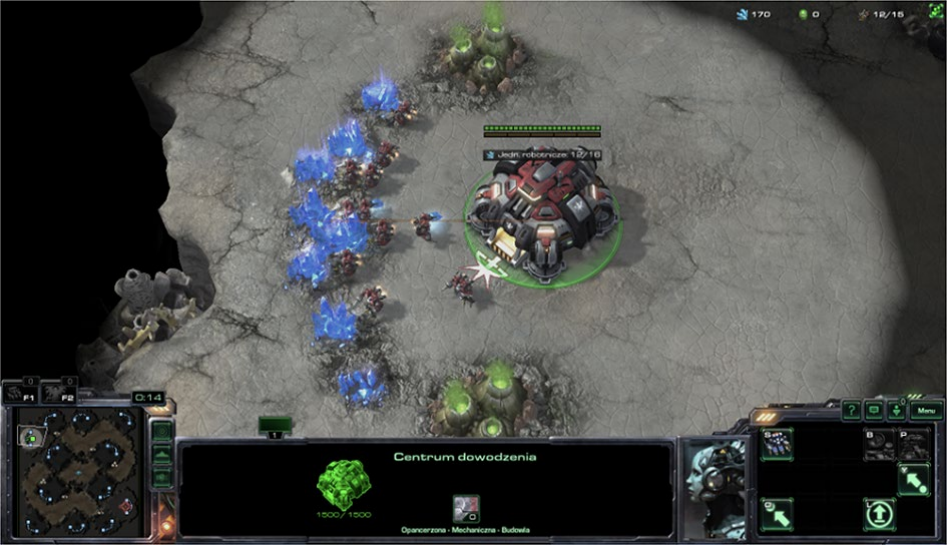
Screenshot of the StarCraft II (©2022 Blizzard Entertainment, Inc.) match played by one of the participants.

Participants underwent StarCraft II gaming sessions (Fig. 2) in controlled laboratory settings with the prohibition of playing outside of the laboratory. Before the first StarCraft II game, each participant underwent an introductory training session with the StarCraft II coach designed to familiarize participants with the core concepts of the game and basic gameplay mechanics.The StarCraft II training in the presented study consisted of 30 h, lasting between 3 and 4 weeks with a minimum duration of 5 h and a maximum duration of 10 h per week. As mentioned before participant were assigned to 2 different groups, “Variable” and “Fixed”. Both groups played all the matches (30 h) as Terran fraction. The subjects in the “Fixed” group always played against Terran fraction and with “Economic Focus” strategy, where in the “Variable” group participants played against different fractions and with different strategies. It is important to mention that players in both groups always played against Artificial Intelligence (AI). The game difficulty varied across 8 levels. Online platform software recorded the number of losses (−1) and wins (+ 1), and difficulty was increased by one each each time the total passed the threshold of multiple of 4. When the total dropped below the threshold the difficulty decreased. Training was carried out using a dedicated desktop PC running Windows 7 (professional edition, 64-bit operating system) equipped with a dedicated graphics card (NVIDIA GeForceGTX 770), 8 GB of RAM, and a 24′′LED display which allowed to play at the high graphic quality (1920 × 1080 pixels resolution, 60 Hz). The participants played the game using a mouse, keyboard and headset.

### Telemetric data from StarCraft II

Telemetric variables were obtained from StarCraft II Replays, using sc2reader (retrieved from https://github.com/ggtracker/sc2reader) and PACanalyzer (retrieved from https://github.com/Reithan/PACAnalyzer), which are Python libraries to extract information from various StarCraft II resources. The mentioned libraries allowed us to extract replay details, such as information on the used map, length of the match, game type, etc., but also more specific data such as (1) player details, (2) unit selection and hotkeys events, (3) resource transfers and requests, (4) unfiltered unit commands such as attack, move, train, built, etc., (4) camera movements. During the study, we collected a total of 1980 StarCraft II replays from 22 participants [each participant produces approximately 90 (SD = 17.5) matches during the training process]. After initial extraction, we filtered matches that lasted longer than 60 min or shorter than 1 min, assuming that these are the results of a computer or user error (such as starting the wrong type of match). Then we have calculated the mean length of the matches for each of the participants. The next filter cut matches longer and shorter than the mean length of played matches ± 1 standard deviation. Telemetric data was analyzed from a total of 1519 matches. Then it is important to mention that game proficiency does not depend only on one specific variable. We decided to focus on basic variables, which at the same time are very sensitive to player’s skills development (Thompson et al., 2017).

1. **Action per minute (APM)**, which is the average number of actions performed per minute in the game and can be interpreted as cognitive, motor, and decision-making speed. APM is the most popular variable used to show how skilled the player is, but only to a certain extent. One source suggests that in average professional players (high league) have higher number of APMs (around 267) when compared to lower-league players (around 60), what could emphasize the skill learning process^28^. As our participants were able to play only for a 30-h period, the increase in APM perfectly reflects the process of gaining the ability to play StarCraft II.
2. **Perception Action Cycle (PAC) Action latency**, where one PAC begins with a attentional switch and ends with the next attentional switch. PAC Action Latency is the average latency from the onset of a PAC to the first undertaken action in that PAC measured in real-time seconds. While the first matches of the participants were played only in one observed region (PAC = 1), along with gaining experience in the game, the participants were able to task-switch extremely quickly between many distant and independent places on the map.
3. **HS (hotkeys) usage**, the average number of hotkey presses per minute in each game, where each of these actions represents an automated selection of multiple units or buildings^25^.

Choosing this variable was based on its importance for players to play in more efficient way, being able to switch between tasks faster and therefore the usage of hotkeys, especially for novices, is necessary to significantly improve their performance.

All three of the chosen variables are characterized by a low starting score, resulting from zero experience of playing, and a regular increase with the subsequent matches played (see Supplementary Materials, Figs. S1, S2 and S3).

It should be noted that although all players showed the same normal trends in development in the discussed variables, the intensity of those increases largely depends on the individual capabilities of the player. Selected variables allow for the observation of the acquisition of abilities at the general level, at the group level, and at the level of individual player achievements. The process of aggregation raw data to individual player observations starts with calculating a number of hotkeys, PACs action latencies and APMs for each player and each match, then computing the means of each variable into a single mean per player (one observation per player).

### Magnetic resonance imaging (MRI)

#### MRI image acquisition

All magnetic resonance (MR) images were collected using a GE Discovery MR750w 3 T MRI scanner (CNS Lab, IBBE PAS, Warsaw, Poland) before RTS training. The MRI scanner was equipped with an 8-channel phased-array head coil. Foam padding was used around the head of the participants to provide comfort and minimize head movements. Subjects were asked to remain still and not fall asleep, while maintaining a comfort level as high as possible.

The spin-echo diffusion weighted echo planar imaging (DW_EPI) sequence was performed with TR = 16,000 ms, TE = 100 ms, flip angle = 90, and voxel size = 2 × 2 × 2 mm^3^. We used AP (anterior–posterior) and PA (posterior-anterior) phase coding directions, 40 volumes with a b-value of 1000 s/mm^2^ for AP, 40 volumes with a b-value of 1000 s/mm^2^ for PA, along with 4 images with applying no diffusion gradient (b-value = 0) for both AP and PA (eight b0 images in total).

#### Diffusion data preprocessing

The entire diffusion data preprocessing procedure was performed in FSL 6.0.4^29^ (FMRIB Software Library, http://www.fmrib.ox.ac.uk/fsl/). First, the B0 images were extracted from the NIfTI files and combined into a single-volume image. We used FDT FMRIB Diffusion Toolbox for the next steps of preprocessing diffusion images^30,31^. Topup tool was used to estimate and correct the susceptibility-induced field distortions^32^. Then, head motions and distortions produced by the gradient coils were reduced using the eddy current correction^33^. The Brain Extraction Tool (BET) was used to isolate and remove nonbrain tissue with a fractional intensity threshold of 0.3. The DTIFIT command was then used to calculate the diffusion tensors and fractional anisotropy (FA). The FA images for all participants were registered to the MNI standard space (FMRIB58_FA_1mm template) using FSL’S FLIRT^34,35^ and FNIRT^36^ tools.

### MRI data analysis

All steps for the diffusion data analysis of both approaches were performed with FSL 6.0.4^29^ (FMRIB Software Library, http://www.fmrib.ox.ac.uk/fsl/).

**Figure 3:**
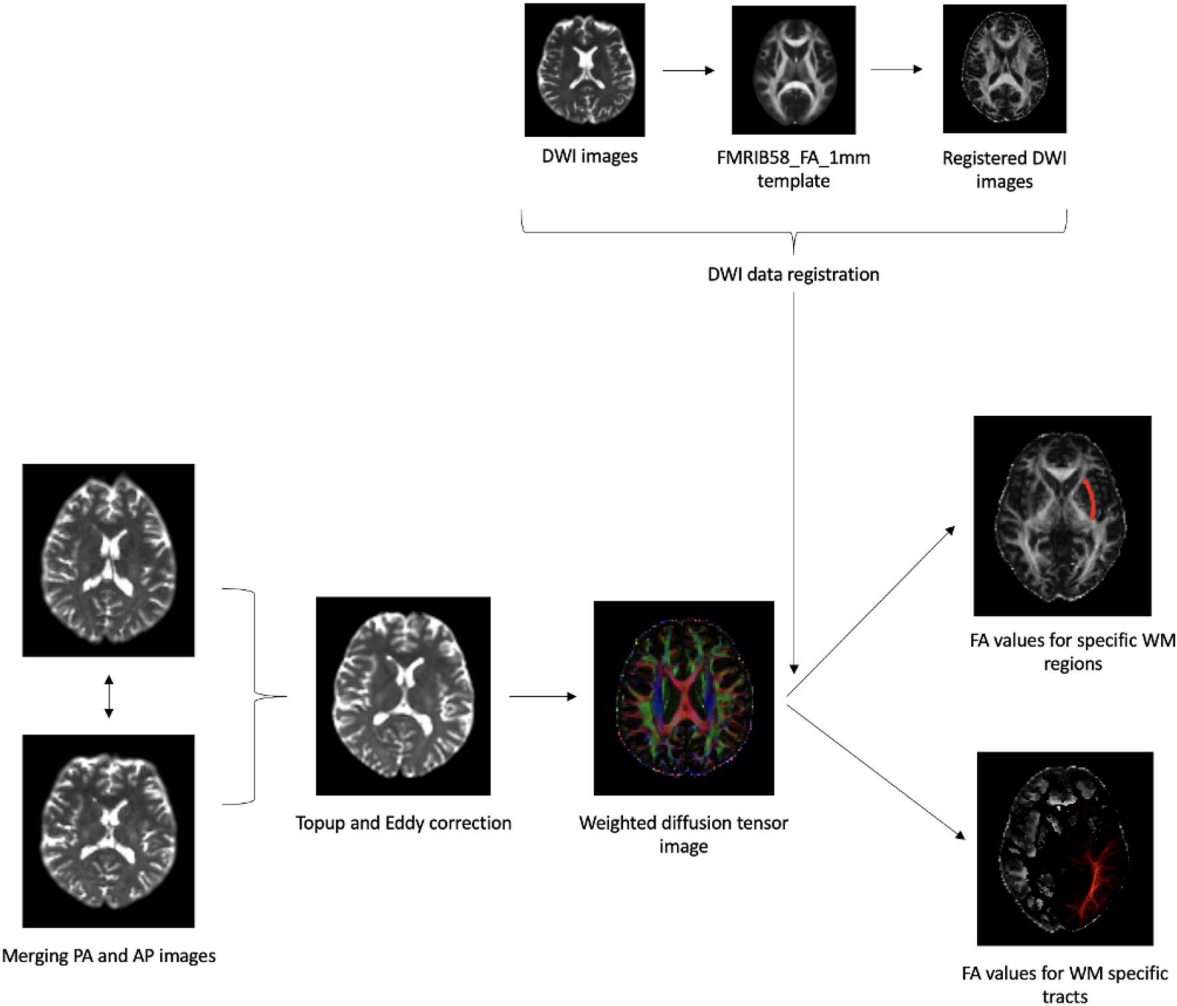
Graphic presentation of the MRI diffusion data processing steps. *PA* posterior–anterior, *AP* anterior–posterior, *DWI* diffusion weighted imaging, *FA* fractional anisotropy, *WM* white matter.

For WM regions (no tractography) the masks were created using the JHU-ICBM DTI 81 White Matter atlas (Mori et al., 2005). Then FA metrics for each person and each region of interest were calculated with a threshold of 10%^10^. For the probabilistic tractography approach, Bedpostx tool^30,31^ was run to build and estimate the distributions of diffusion parameters at each voxel and model the crossing fibers using Markov Chain Monte Carlo sampling. Probabilistic tracking was performed using the Probtrackx tool^30,31^, with 5000 samples taken for each input voxel with a 0.2 curvature threshold. The FA values for each extracted pathway were calculated with a threshold of 10% to exclude low-probability voxels^10^. Figure 3 represents the steps for processing the diffusion data.

### White matter regions and tracts selected for analysis

**Figure 4:**
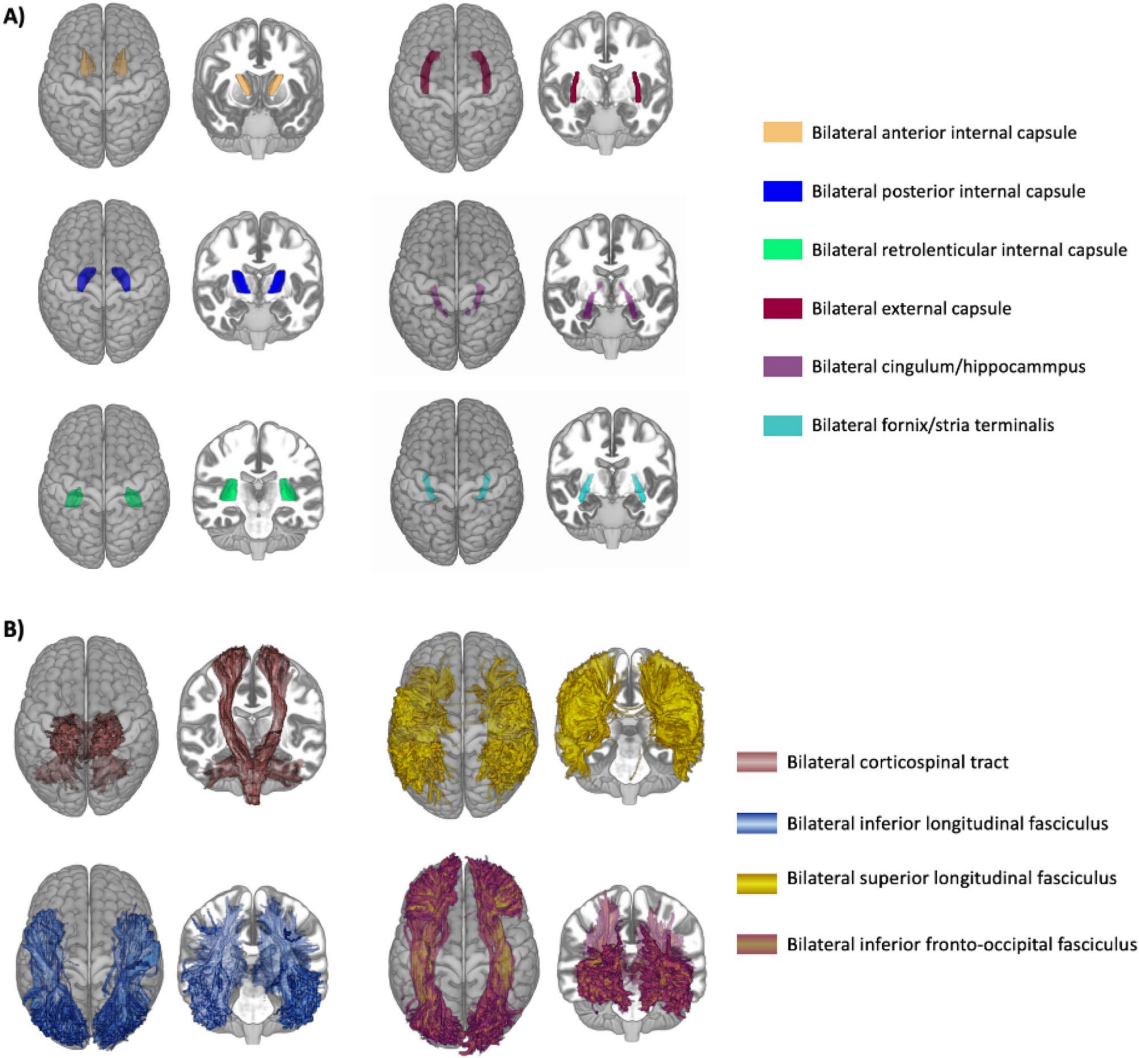
Graphical representation of the brain regions chosen for analysis. (**A**) Represents the chosen WM regions and (**B**) chosen tracts for the probabilistic tractography-based approach.

The white matter regions of interest were chosen based on the literature described in the Introduction section and presented in Fig. 4. For the approach based on the extraction of FA values from the WM regions (without tractography) we focussed on the bilateral anterior limb of the internal capsule (ALIC), bilateral posterior limb of the internal capsule (PLIC), bilateral retrolenticular limb of the internal capsule (RLIC), bilateral external capsule (EC), bilateral cingulum/hippocampus (CG/HIP) and bilateral fornix stria terminalis (FX/ST). The external capsule and parts of the internal capsule were chosen based on the research by Kowalczyk-Grębska et al.^9^. The cingulum and fornix areas are based on the study by Ray et al.^15^.

For the probabilistic tractography approach, motor and visual pathways were chosen: bilateral corticospinal tract (CST), bilateral superior longitudinal fasciculus (SLF), bilateral inferior longitudinal fasciculus (ILF), and bilateral inferior fronto-occipital fasciculus (IFOF). The tracts were chosen based on the study by Zhang et al.^10^ where the results revealed significant differences in these tracts between VGPs and amateurs.

### Statistical analysis

To evaluate the association between white matter microstructure and game learning, correlation analyzes were performed using Jamovi software, version 2.3.12 (retrieved from https://www.jamovi.org, 2001) and RStudio software, version 2022.02.0 + 443, (retrieved from http://www.rstudio.com/, 2020).

### Ethics statement

The study involving human participants as reviewed and approved by Komisja ds. Etyki Badań Naukowych Wydziału Psychologii w Warszawie (Ethics committee of the Department of Psychology at University of Social Sciences and Humanities, number: 38/2018). The participants provided written informed consent to participate in this study.

## Results

### Association between FA in WM regions and StarCraft II game indicators.

The Spearman coefficient correlation analysis was performed to examine the association between StarCraft II performance measures and white matter integrity as FA values in WM regions.

**Figure 5:**
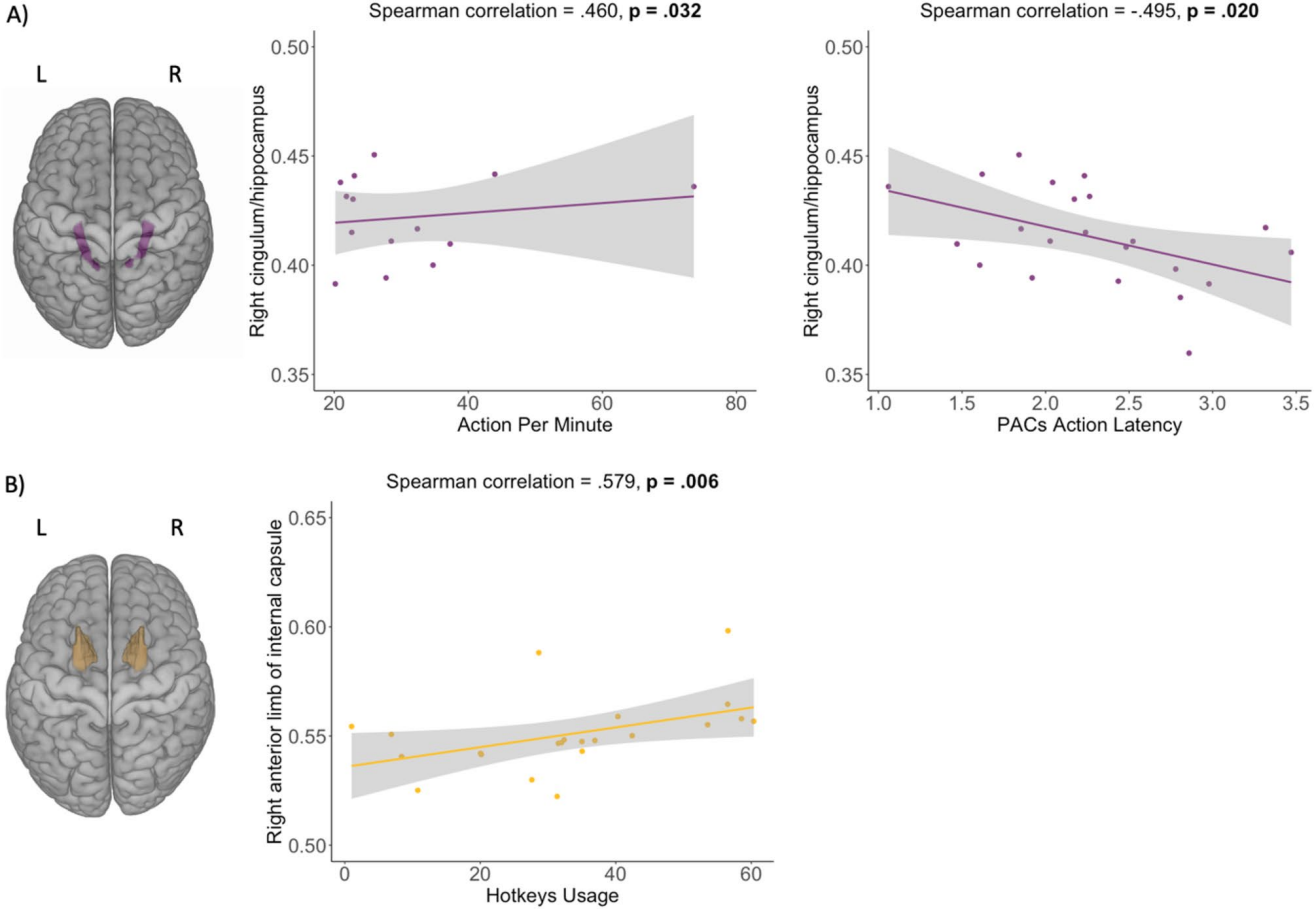
*ROIs* regions of interest, *L* left, *R* right. Predefined ROIs (right cingulum/hippocampus (**A**)) and scatter plots representing the significant associations between FA values in ROIs and StarCraft II skill acquisition indicators. The purple color represents the results for cingulum/hippocampus, and yellow represents the anterior limb of the internal capsule. The results presented in the figure are without the FDR correction.

We correlated all previously described ROIs with Action per Minute, PACs Action Latency, and Hotkeys Usage (Table 1). We found that the FA value in the right cingulum/hippocampus (Fig. 5A) was positively associated with Action Per Minute (r = 0.460, p = 0.032) and negatively associated with PACs latency (r = − 0.495, p = 0.02). The value of FA in the right anterior limb of the internal capsule (Fig. 5B) was positively associated with the average total use of hotkeys in StarCraft II (r = 0.570; p = 0.006). All correlations have also passed pairwise comparison using the False Discovery Rate (FDR) adjustment method (p < 0.05).

**Table 1:** Results of correlations between FA in the WM regions and StarCraft II performance indicators.

Brain regions	Action per minute		PACs latency		Average hotkeys usage	
	*rho*	*p*	*rho*	*p*	*rho*	*p*
CG/HIP L	0.377^†^	0.085	− 0.392^†^	0.072	− 0.134	0.551
CG/HIP R	0.460*	**0.032**	− 0.495*	**0.020**	0.295	0.182
FX/ST L	00.033	0.884	− 0.022	0.924	0.375	0.086
FX/ST R	0.234	0.292	− 0.281	0.205	0.315	0.154
ALIC L	0.274^†^	0.217	− 0.278	0.209	0.381^†^	0.081
ALIC R	0.317	0.151	− 0.357	0.103	0.579**	**0.006**
PLIC L	0.182	0.415	− 0.143	0.524	0.162	0.469
PLIC R	0.277	0.211	− 0.136	0.544	0.225	0.312
RLIC L	0.212	0.343	− 0.149	0.508	0.300	0.175
RLIC R	0.162	0.469	− 0.137	0.541	0.264	0.235
EC L	0.276	0.213	− 0.311	0.159	0.169	0.451
EC R	0.381^†^	0.081	− 0.382^†^	0.080	0.126	0.575

The results presented in the table are without the FDR correction.

*L* left, *R* right, *CG/HIP* cingulum/hippocampus, *FX/ST* fornix/stria terminalis, *ALIC* anterior limb of internal capsule, *PLIC* posterior limb of internal capsule, *RLIC* retrolenticular limb of internal capsule, *EC* external capsule, *PACs* perception action cycles.

** *p* < 0.01; * *p* < 0.05; ^†^ *p* < 0.10. Significant results are in bold.

### Associations between FA along WM tracts and StarCraft II game indicators

To explore the associations between FA within white matter tracts and StarCraft II performance measures, we performed a Spearman coefficient correlation analysis. We correlated FA values along all previously described tracts with Action per Minute, PACs Action Latency, and averaged Hotkeys Usage (Table 2).

**Table 2:** Correlation results between FA along the tracts and StarCraft II performance indicators.

Tracts	Action per minute		PACs per minute		Average hotkeys usage	
	*rho*	*p*	*rho*	*p*	*rho*	*p*
CST L	0.003	0.992	− 0.033	0.884	− 0.089	0.694
CST R	0.041	0.856	− 0.074	0.743	− 0.093	0.679
SLF L	− 0.041	0.856	− 0.011	0.964	− 0.138	0.538
SLF R	− 0.029	0.900	− 0.004	0.988	− 0.076	0.736
IFOF L	0.397^†^	0.068	− 0.403^†^	0.064	0.240	0.281
IFOF R	0.286	0.196	− 0.318	0.149	0.165	0.460
ILF L	0.457*	**0.034**	− 0.474*	**0.027**	0.252	0.256
ILF R	0.359	0.102	− 0.355	0.105	0.221	0.322

The results presented in the table are without the FDR correction.

*L* left, *R* right, *CST* corticospinal tract, *SLF* superior longitudinal fasciculus, *ILF* inferior longitudinal fasciculus, *IFOF* inferior fronto-occipital fasciculus, *PACs* perception action cycles.

* p < 0.05; ^†^p < 0.10. Significant results are in bold.

**Figure 6:**
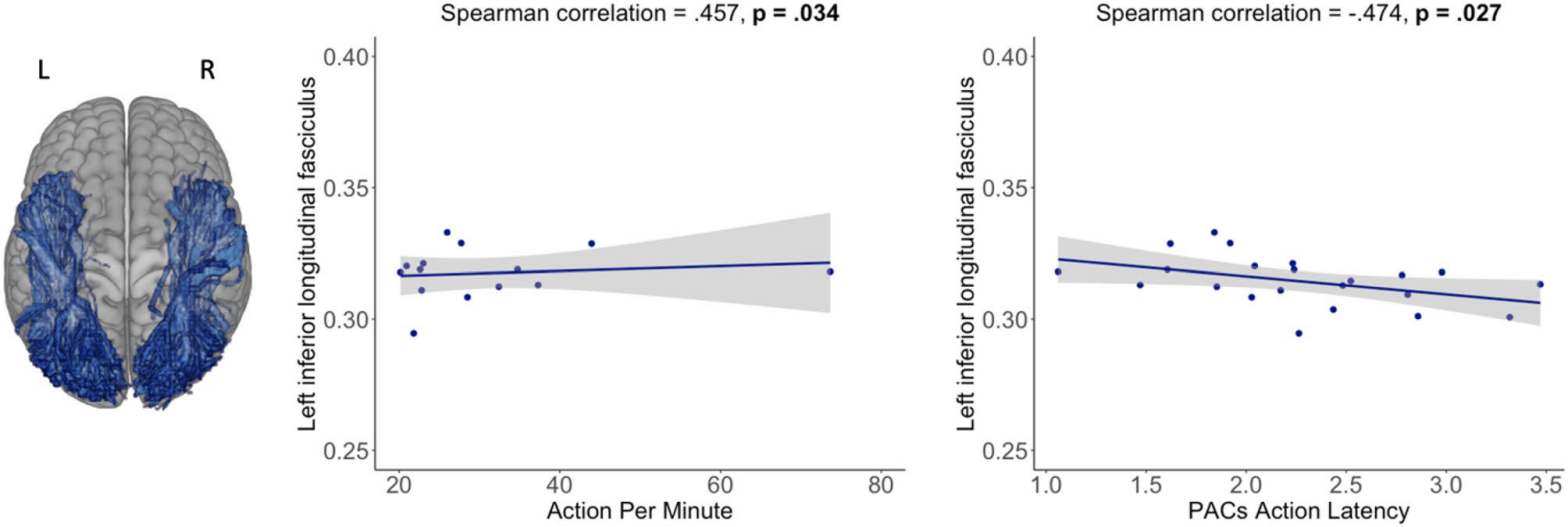
*ROIs* regions of interest, *L* left, *R* right. Predefined ROIs (left inferior longitudinal fasciculus) and scatter plots representing the significant association between FA values along the left inferior longitudinal fasciculus and StarCraft II skill acquisition indicators. The results presented in the figure are without the FDR correction.

We found that the FA value in the left inferior longitudinal fasciculus (Fig. 6) was positively associated with Action Per Minute (r = 0.457, p = 0.034) and negatively with the PACs latency (r = − 0.474, p = 0.02). Both of these correlations have also passed a pairwise comparison using the FDR adjustment method (p < 0.05).

The results revealed significant correlations between white matter integrity (FA) in the selected regions (right anterior limb of internal capsule, right cingulum/hippocampus) and the tracts (left inferior longitudinal fasciculus), and StarCraft II performance outcomes. This indicates that higher white matter integrity in regions of interest and tracts is associated with better StarCraft II performance.

## Discussion

In the presented study, we aimed to find associations between the outcome of the training with a complex video game and the pre-training white matter microstructure characterized with two methods (1) FA within white matter regions and (2) FA along white matter tracts using probabilistic tractography. Studies using video games as a training tool usually did not focus on individual differences in the learning process. However, some research emphasized that neural factors may play an important role in predisposition for complex task learning, including video games^9,15,20,37,38^. Our study indicates that white matter microstructure plays an important role as a factor predisposing for acquisition of complex skills while playing VGs. The FA within the right cingulum region near the hippocampus turned out to be positively correlated with Actions Per Minute (APM) and Perception Action Cycles (PACs) latency measures derived from the StarCraft II game. Previously, the FA value within this region was found to be a significant predictor of strategy game learning (Sushi-Go-Round), suggesting that playing such games can engage memory and cognitive control processes^15^ (Ray et al., 2018). Furthermore, the cingulum hippocampus region is considered a part of the core memory circuitry in amnestic mild cognitive impairment (aMCI), where research shows that people with MCI tend to have lower FA within this region^39^. In the same study^39^ across groups (aMCI and healthy controls), the analysis showed that the FA within cingulum hippocampus is positively associated with The Symbol Digit Modalities Test (SDMT), which assesses psychomotor speed^40^.

In our study, the described above WM regions seem to have a significant impact on the selected StarCraft II indicators. The greater number of actions represented by APMs is associated with a better understanding of the structure of the game itself and the recall of some basic strategies, which should be performed at specific moments of the gameplay. PAC action latency is directly related to remembering and taking (usually adequate) actions in many remote areas, which strain both memory and executive functions. The structures identified in previous research thus seem to actually influence both: the course of the match and the development of the skill of playing video games. It should be emphasized that the use of accurate game indicators that allow controlling the process of acquiring a new skill and well illustrate the level of player advancement is rather a new method and referring to other studies is extremely difficult.

A positive correlation was also found between FA within the right anterior limb of the internal capsule and the average usage of hotkeys. This part of the internal capsule carries thalamic and brainstem fibers from the prefrontal cortex areas and has been found to be associated with cognition processing and decision making^41–43^. It is also placed near the gray matter regions, caudate nucleus, and lenticular nucleus, which were significantly associated with the StarCraft II performance outcomes^9^. It was also found that FA in the inferior anterior limb of the internal capsule was related to a set-shifting ability that reflects cognitive flexibility^44^. The use of hotkeys that are completely new for our participants (they are in no way consistent with the keyboard shortcuts used daily) reflects the ability to learn, adapt, and fast thinking in the response to the new environment. From a certain point of advancement, the use of hotkeys is a crucial skill that allows players to perform actions faster and therefore react faster. It is also worth mentioning that the acquisition of this skill was in a way forced by the increasing level of difficulty in the game^25^.

The probabilistic tractography analysis revealed that only the FA along the left inferior longitudinal fasciculus was significantly related to two of the chosen StarCraft II performance indicators, APM and PACs Action Latency. The available literature suggests that the inferior longitudinal fasciculus is primarily involved in visual functions, such as processing and modulating visual cues and visually guided decisions and behavior^45,46^. The selection of tracts for our analysis was based on the cross-sectional study, in which the FA value in all selected tracts differentiated between players and non-players^10^. Once again, both selected indicators can be understood as related to decision-making. Most importantly, as visual stimuli are the most important in the game, the entire process is based on visual processing, which is consistent with previous investigations.

It is worth highlighting that we aimed to establish an overall association between pre-training white matter metrics and game performance, but we did not focus on the differences between fixed and variable groups. Although it is highly possible that the highest improvement in skill acquisition could be seen in the first 30 h of gameplay, analysis including the full process of playing (60 h), a larger sample size, and group differences in the applied model of training (fix and variable) should be taken into consideration in the future.

Additionally, we would like to address few other limitations of the presented study. As mentioned above, the analysis only focused on the associations between pre-training white matter metrics and game performance. However, in the future studies it should be considered exploring comprehensively VGs and the variables from the game with regards to their link to conventional psychological test-metrics. Also, the issue of telemetric variables should be taken into account. As mentioned in the Methods section, we decided to select three variables that are able to accurately illustrate the development of RTS players at the beginner and intermediate levels of experience^25^. Hotkeys usage and Action Per Minute turned out to be strongly correlated with each other, which is a result from the game structure. With experience, players learn new hotkeys, allowing them to increase their number of actions per minute. Then, it is important to mention that StarCraft II is a complex game, and it is impossible to establish a precise process of skill development using only three indicators. However, the indicators that we chose clearly to show the overall process of learning a new skill, such as playing an RTS game. The same indicators are often used by players themselves to monitor their own progress.

## Conclusions

This work presents an insight into the process of complex task learning and the importance of the pre-training white matter individual differences in its efficiency. The results are inline with previous work^9,10,15,37^ demonstrating the importance of brain characteristics in the effectiveness of complex skill learning, but also extend the available work by using two different approaches for analyzing WM microstructure.

In this study, the white matter regions and tracts that were significantly associated with indicators of StarCraft II performance are involved in psychomotor functions^39^, set shifting ability42, and visually guided decisions and behavior^45,46^. It is worth mentioning that previous studies mostly used rather simple video games, such as Space Fortress^37^ or Sushi-Go-Round^15^, where in our study we can see similar patterns yet on a more advanced RTS game. Finally, presented results indicate that people with specific neural predispositions can achieve better scores in complex task-learning processes. This suggests the importance of neuroimaging studies, with the focus on the white matter, in predicting the outcomes of longitudinal studies using interventions. The results may also find its application in identifying the neural attributes of eSports players’ success.

## Supplementary Information

Supplementary Information.

## Data Availability

The datasets generated during and/or analyzed during the current study are available from the corresponding author on reasonable request.
