# Author Correction: Association between real-time strategy video game learning outcomes and pre-training brain white matter structure: preliminary study

**DOI:** 10.1038/s41598-023-27873-0

**Published:** 2023-01-13

**Authors:** Paulina Lewandowska, Natalia Jakubowska, Nikodem Hryniewicz, Rafał Prusinowski, Bartosz Kossowski, Aneta Brzezicka, Natalia Kowalczyk-Grębska

**Affiliations:** 1 Institute of Psychology, Jagiellonian University, Romana Ingardena 6, 30-060 Krakow, Poland; 2 Faculty of Psychology, SWPS University of Social Sciences and Humanities, Chodakowska 19/31, 03-815 Warsaw, Poland; 3 CNS Lab, Nalecz Institute of Biocybernetics and Biomedical Engineering, PAS, Księcia Trojdena 4, 02-109 Warsaw, Poland; 4 Laboratory of Brain Imaging, Nencki Institute of Experimental Biology, PAS, Ludwika Pasteura 3, 02-093 Warsaw, Poland; 5 Polish-Japanese Academy of Information Technology, Koszykowa 86, 02-008 Warsaw, Poland

Correction to: *Scientific Reports* 10.1038/s41598-022-25099-0, published online 01 December 2022

The original version of this Article contained an error in the Funding section.

“This study was supported by the Polish National Science Center, grant number: 2016/23/B/HS6/03843. N.K-G was supported by the Foundation of Polish Science (FNP) and the Kosciuszko Foundation.”

now reads:

“This study was supported by the Polish National Science Center, grant number: 2016/23/B/HS6/03843. N.K-G was supported by the Foundation of Polish Science (FNP) and the Kosciuszko Foundation. Open access was funded by SWPS University Research Development.”

The original Article has been corrected.

