# Supplementary material for: Association between real-time strategy video game learning outcomes and pre-training brain white matter structure: preliminary study
Source: Sci Rep. 2022 Dec 1;12:20741. doi: 10.1038/s41598-022-25099-0 (PMC9715544; doi:10.1038/s41598-022-25099-0)
Supplement: Supplementary file 1 — Supplementary Information. [file 41598_2022_25099_MOESM1_ESM.docx]

**Supplementary materials**


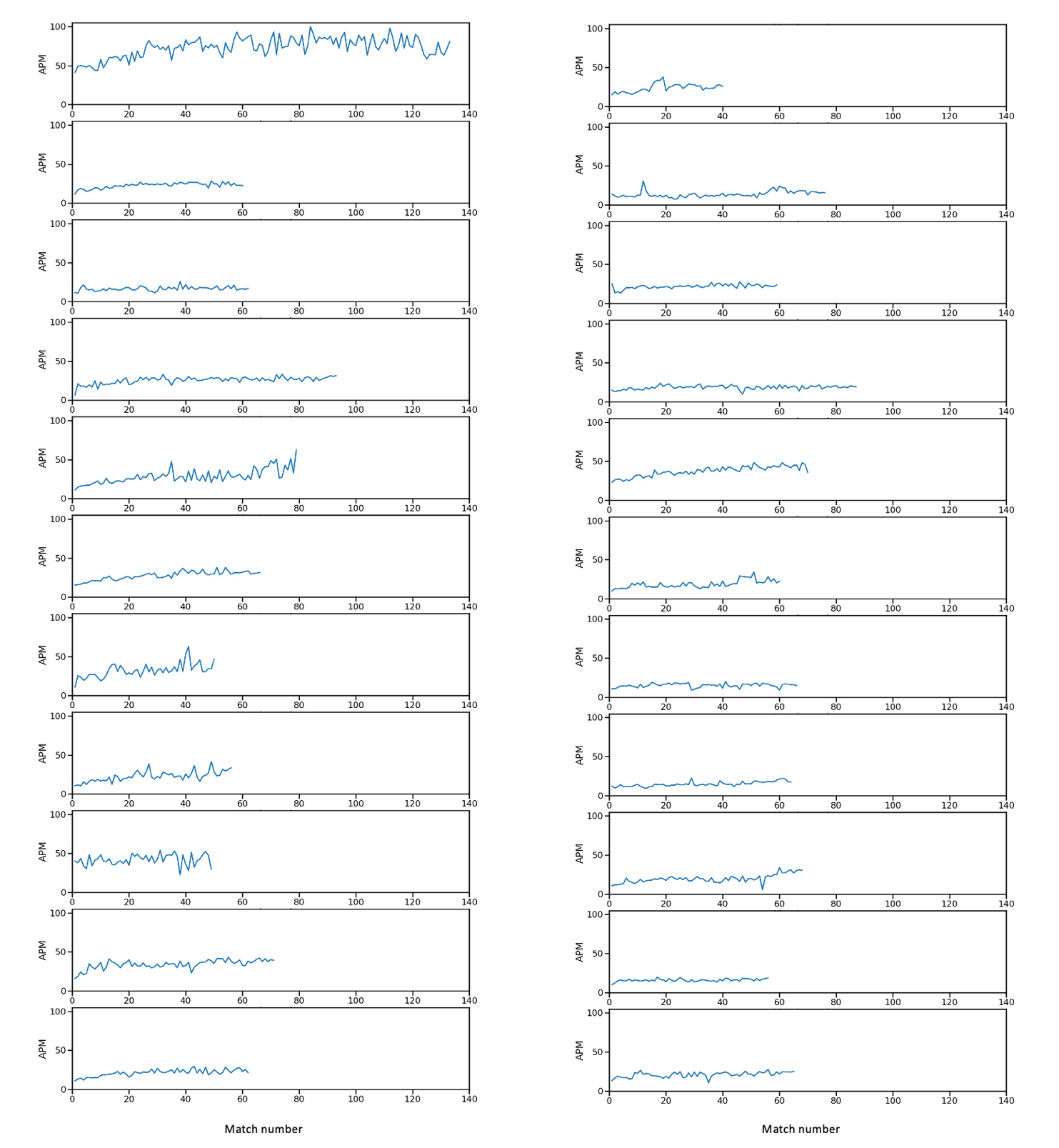


**Supplementary figure 1 |** Figure represents learning curves for each participant prepared on the raw data. On the X axis are the number of played matches whereas on Y axis APM indicator.

APM – Action Per Minute.


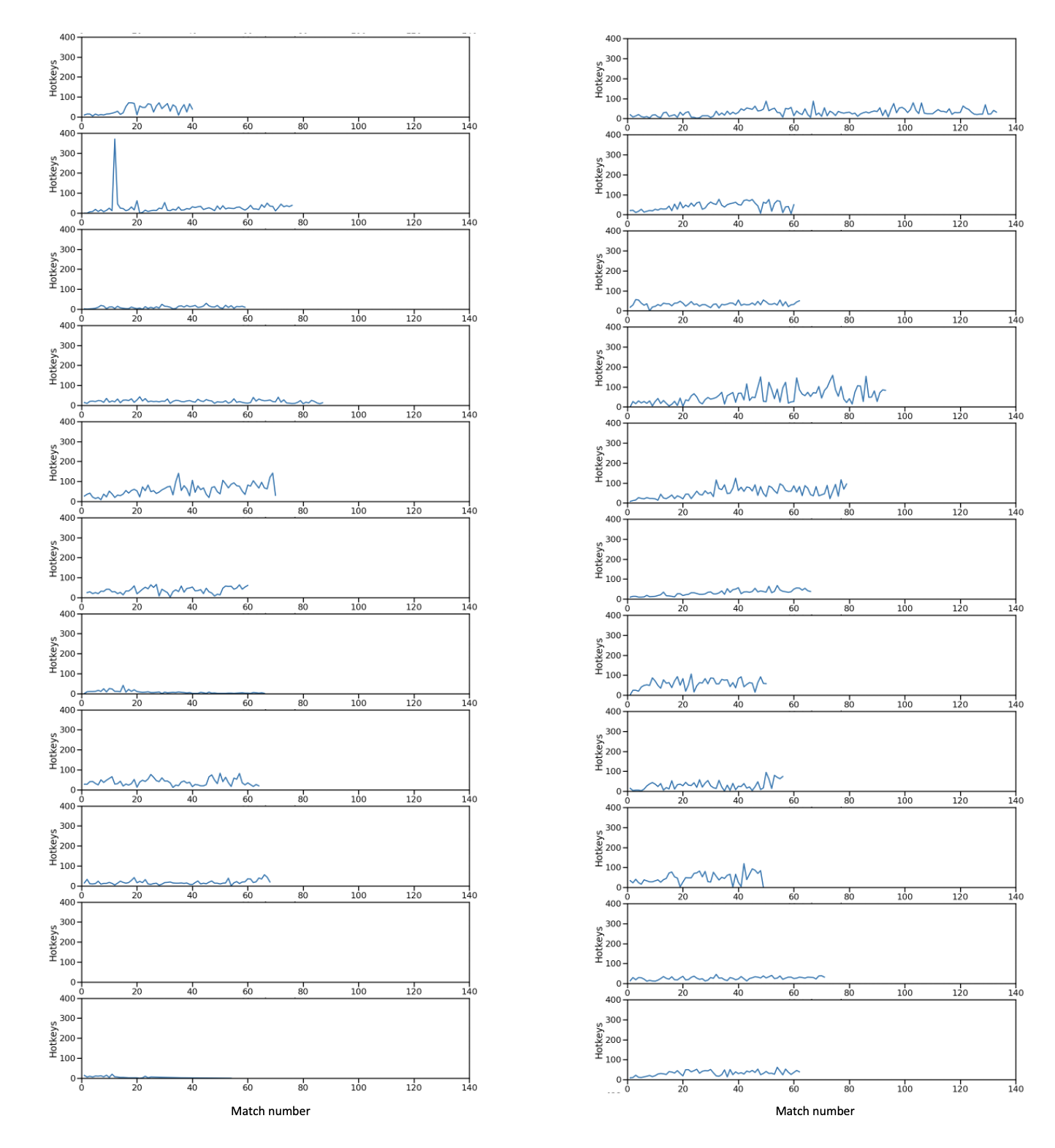


**Supplementary figure 2 |** Figure represents learning curves for each participant prepared on the raw data. On the X axis are the number of played matches whereas on Y axis hotkeys variable.


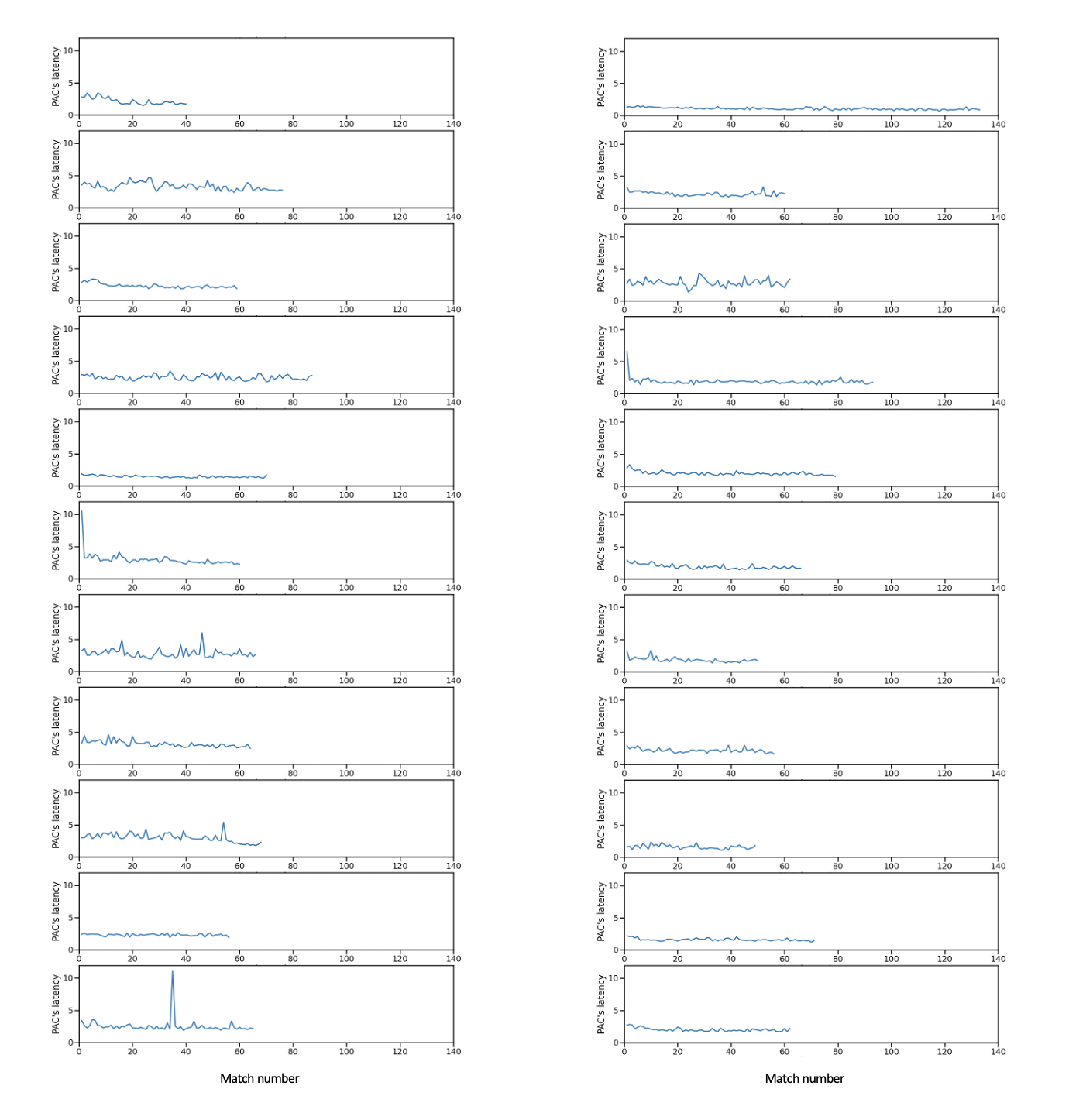


**Supplementary figure 3 |** Figure represents learning curves for each participant prepared on the raw data. On the X axis are the number of played matches whereas on Y axis PAC’s latency variable measured in real-time seconds. PAC’s – Perception Action Cycles
